# Egg load dynamics and the risk of egg and time limitation experienced by an aphid parasitoid in the field

**DOI:** 10.1002/ece3.1023

**Published:** 2014-04-11

**Authors:** Christine Dieckhoff, Julian C Theobald, Felix L Wäckers, George E Heimpel

**Affiliations:** 1 Department of Entomology & Wildlife Ecology, University of DelawareNewark, Delaware, 19716; 2 Lancaster Environment Centre, Centre for Sustainable Agriculture, Lancaster UniversityLancaster, LA1 4YQ, UK; 3 Department of Entomology, University of MinnesotaSt Paul, Minnesota, 55108

**Keywords:** *Aphis glycines*, *Binodoxys communis*, biological control, egg load, soybean aphid

## Abstract

Insect parasitoids and herbivores must balance the risk of egg limitation and time limitation in order to maximize reproductive success. Egg and time limitation are mediated by oviposition and egg maturation rates as well as by starvation risk and other determinants of adult lifespan. Here, we assessed egg load and nutritional state in the soybean aphid parasitoid *Binodoxys communis* under field conditions to estimate its risk of becoming either egg- or time-limited. The majority of female *B. communis* showed no signs of egg limitation. Experimental field manipulations of *B. communis* females suggested that an average of 4–8 eggs were matured per hour over the course of a day. Regardless, egg loads remained constant over the course of the day at approximately 80 eggs, suggesting that egg maturation compensates for oviposition. This is the first case of such “egg load buffering” documented for a parasitoid in the field. Despite this buffering, egg loads dropped slightly with increasing host (aphid) density. This suggests that egg limitation could occur at very high host densities as experienced in outbreak years in some locations in the Midwestern USA. Biochemical analyses of sugar profiles showed that parasitoids fed upon sugar in the field at a remarkably high rate. Time limitation through starvation thus seems to be very low and aphid honeydew is most likely a source of dietary sugar for these parasitoids. This latter supposition is supported by the fact that body sugar levels increase with host (aphid) density. Together, these results suggest that fecundity of *B. communis* benefits from both dynamic egg maturation strategies and sugar-feeding.

## Introduction

Both egg limitation and time limitation can lead to missed reproductive opportunities in insect parasitoids and herbivores. Females are therefore predicted to balance the risk of becoming egg- or time-limited to maximize lifetime fecundity (Minkenberg et al. 1992; Rosenheim 1996; Heimpel et al. 1998; Rosenheim et al. 2008). The extent to which female parasitoids and herbivores are egg- versus time-limited in the field is not very well known, although field egg loads of a handful of species have been studied (reviewed by Heimpel and Rosenheim 1998; Heimpel and Casas 2008; Cummins et al. 2011). These studies have shown variable rates of egg limitation, but most have not assessed the risk for time limitation (as estimated by life expectancy) (but see Heimpel et al. 1997; Lee and Heimpel 2008; Rosenheim et al. 2008; Berger et al. 2012). Here, we use egg load counts and biochemical assays of the nutritional status of individual females of the aphid parasitoid *Binodoxys communis* (Gahan) (Hymenoptera: Braconidae) collected in the field to estimate risks of egg limitation and time limitation in this species.

Egg limitation is mediated by oviposition, egg maturation, and resorption rates (Richard and Casas 2012). In synovigenic insects (i.e., those that mature eggs as adults; Jervis et al. 2001), egg maturation rates may respond to internal conditions such as egg load or external factors such as host density in ways that minimize the risk of egg limitation. An increase in the egg maturation rate in response to low egg loads has been documented in several parasitoid species (Rivero-Lynch and Godfray 1997; Wu and Heimpel 2007), including *B. communis* (Dieckhoff and Heimpel 2010). In addition, Casas et al. (2009) have shown that host contact alone can increase the ecdysone levels in the parasitoid *Eupelmus vuilletti* resulting in an increase in egg load. Together, these phenomena are termed “dynamic egg maturation rates,” and they can provide a buffer to egg load, thus lowering the risk of egg limitation in parasitoids.

While egg maturation rates may influence egg limitation, longevity influences the extent to which insects become time-limited. To maximize longevity in the field, many parasitoids require a carbohydrate source such as nectar or hemipteran honeydew (Wäckers 2005). Hemipteran honeydew is thought to be the dominant nonhost carbohydrate source for insects in highly simplified agricultural settings (Wäckers 2005; Wäckers et al. 2008; Tena et al. 2013). However, some honeydews have relatively low nutritional value (Wäckers et al. 2008).

The purpose of this study was to assess egg load and nutritional status in the aphid parasitoid *Binodoxys communis* under field conditions in order to estimate its risk of egg limitation and time limitation in the field. This parasitoid species is native to Asia and has been introduced into the Midwestern USA as a biological control agent against the soybean aphid, *Aphis glycines* Matsumura (Hemiptera: Aphididae) (Ragsdale et al. 2011) and into Hawaii as a biological control agent of the cotton aphid, *Aphis gossypii* Glover (Hemiptera: Aphididae) (Acebes and Messing 2013a,b2013b). *B. communis* is a synovigenic parasitoid that does not engage in host feeding and is specialized on *A. glycines* and close relatives (Wyckhuys et al. 2008a; Desneux et al. 2009a, 2012; Dieckhoff and Heimpel 2010; Acebes and Messing 2013a). Previous research has shown that feeding on a carbohydrate source has no influence on egg load but significantly increases longevity in *B. communis* (Wyckhuys et al. 2008b; Dieckhoff and Heimpel 2010). Furthermore, *B. communis* maintains a high egg load through egg maturation in response to decreases in egg load in the laboratory (Dieckhoff and Heimpel 2010). We hypothesize that this mechanism helps attenuate egg limitation in the field.

## Materials and methods

### Insects

*Binodoxys communis* was reared on *Aphis glycines*-infested soybean plants in a greenhouse at the University of Minnesota, St. Paul, MN, USA. The *A. glycines* colony was established from aphids collected from a soybean field in St. Paul in 2003 and was reared continuously on soybean plants (Syngenta NK S19-R5) in growth chambers (16:8 h light:dark; 60–80% r.h.; 25 ± 5°C).

The *B. communis* colony originated from a strain collected in the Chinese province of Heilongjiang in 2002 (Wyckhuys et al. 2008a). Releases across the Midwestern USA have been conducted since 2007; however, at the time of this study, there was no evidence that *B. communis* had established in North America (Heimpel et al. 2010; Asplen et al. 2011). Parasitoids were reared in clear plastic storage boxes (62 × 45 × 18 cm; Sterilite®, Townsend, MA) with ventilation windows cut into the long sides and the lid. Windows were covered with a coarse mesh (mesh size: 2 mm) to prevent predators from entering the boxes in the field and a layer of no-see-um mesh (mesh size: 0.6 mm; Quest Outfitters, Sarasota, FL) on the outside of the coarse mesh during parasitoid rearing. Each box contained between six (in 2007) and thirteen (in 2008) 9 × 9 cm square plastic pots containing aphid-infested soybean plants and 16–26 female and twice as many male *B. communis* parasitoids. In 2008, a subsample of 2 pots per rearing container was removed on the morning of each release to estimate the sex ratio of released parasitoids and the number of released female parasitoids.

### Field plots and releases

This study was conducted in soybean fields at the University of Minnesota Agricultural Experiment Station in St. Paul, MN, USA in the summers of 2007 and 2008. The field used in 2007 was 1.02 ha of “cropLAN #RC1992”, and the field used in 2008 was 0.73 ha of “Asgrow #AG1402RR”. *Binodoxys communis* were released weekly into the center of flagged 4 × 4 m sections (“plots”) within the soybean fields from 29 June to 31 August 2007 and 2 July to 27 August 2008. Each plot contained approximately 468 plants, was used for a single release with at least 30 m between individual plots, and was positioned approximately 30 m away from the field edges. Soybean aphid density (host density) within each plot was assessed by destructive sampling of 10 randomly collected soybean plants on the day prior to each release. The mean values of those 10 plant counts provided an estimate of the average per-plant host densities for each plot. In addition, in 2008, 10 randomly collected soybean plants were destructively sampled approximately 2 weeks after each release to assess the average number of first generation *B. communis* mummies on a per-plant and per-plot basis. An estimate of female parasitoid fecundity per plot was then calculated by dividing the estimated number of mummies per plot by the total number of female *B. communis* recovered in each plot. ANOVA was used to assess the difference in host densities over time in 2007 and 2008; a *t*-test was then used to determine differences in densities between the two years of this study. The effects of plot and host density on the estimated number of mummies per plot and female were analyzed using ANCOVA (JMP 8.0.1; SAS Institute, Inc., Cary, NC). Host densities in both years were log_10_-transformed and the estimated numbers of mummies per plot and female in 2008 were (log_10_ + 0.5)-transformed to meet the assumption of homogeneity.

Prior to the first release in both years, fields were treated with the herbicide Roundup© (Monsanto Technology LLC, Bozeman, MT). Daily average temperatures for each day after release were obtained from an NCDC NOAA weather station located at the Minneapolis-St. Paul (MSP) Airport (44°56′N/93°03′W, Bloomington, MN).

Each parasitoid release was performed over a 24-h period each starting between 9 and 10 A.M. on each release date by placing three rearing containers into the center of a plot. Adult parasitoids were collected on the 2 days following each release between 9 A.M. and 4 P.M. or until no parasitoids were recovered for 3 h. Soybean plants in each plot were manually searched for a minimum of 10 min and adult *B. communis* were aspirated, transferred singly into 0.65-mL microcentrifuge tubes (DOT Scientific, Inc., Burton, MI), and immediately put on ice to halt egg maturation and sugar metabolism. Parasitoids caught between 9 and 11 A.M., and 11 A.M. and 4 P.M. will be referred to as “morning” and “afternoon” females, respectively. In addition, a subset of females – referred to as “caged” – collected between 9 and 11 A.M. was transferred individually onto a single aphid-free soybean plant covered with a clear plastic cylinder with no-see-um mesh-covered holes for ventilation (21 × 9.7 cm; Pioneer Plastics, Dixon, KY). These “caged” parasitoids were placed in the shade among field soybean plants for 8 h before they were frozen. The purpose of caging some parasitoids in the field was to compare egg loads of parasitoids foraging freely in the presence of hosts to those deprived of hosts. All parasitoids were stored at −80°C prior to being dissected.

In order to estimate how many parasitoids were released per plot, rearing containers were sealed in the field and returned to the laboratory after each 24-h period, in 2008 only. The number of live parasitoids and the number and fate (emerged, unemerged, or eaten) of *B. communis* mummies in each container were recorded. The number of female parasitoids released per plot was calculated by adding the estimated number of female parasitoids per container in each plot: (Number of emerged mummies - Number of live parasitoids in container) × (1 - Sex Ratio estimate for each box). Recapture rates per plot were calculated by dividing the number of recaptured females by the estimated number of released female *B. communis*.

### Dissections and preparation for sugar analyses

Egg loads of field-collected parasitoids were assessed by dissecting each female under a dissecting microscope at 50× magnification. Ovaries were removed for egg load counts, and parasitoid size was assessed by removing a single hind leg from each individual and measuring the length of the tibia using an ocular micrometer within a dissecting microscope (50× magnification) (see Dieckhoff and Heimpel 2010 for a more complete description of the dissection methods).

Female parasitoids collected in 2008 only were also used for a sugar analysis as described below. After removing their ovaries for the egg load counts, the rest of the insect body was transferred individually into microcentrifuge tubes filled with 40 *μ*L of Ringer's solution and 100% ethanol at a 1:1 ratio and stored at room temperature until analysis.

Effects of hind tibia length, daily average temperature, host density (log_10_-transformed), the number of days after release, and parasitoid group (“morning,” “afternoon” vs “caged”) on egg load were analyzed using multiple linear regression (JMP 8.0.1; SAS Institute, Inc., Cary, NC).

### Egg maturation and oviposition rates

Field egg maturation rates were calculated using linear regressions of egg loads of “morning” and “caged” parasitoids against time for each of the days of collection.

Points along the regression line represent hypothetical egg loads, that is, the number of eggs a female parasitoid matured beyond the baseline value in the morning over the course of a field day in the absence of oviposition. In addition, the difference between the hypothetical egg load and the egg load of each “afternoon” female provides an estimate of the number of eggs laid, that is, the oviposition rate, over the course of a day (Casas et al. 2000; Lee and Heimpel 2008). The effects of the number of days after release and hind tibia length on the number of eggs laid were assessed using ANCOVA (JMP 8.0.1; SAS Institute, Inc., Cary, NC).

### Sugar analysis using high-performance liquid chromatography (HPLC)

To determine the nutritional status of *B. communis*, sugar analyses were performed on 117 female parasitoids collected in 2008. In preparation for analysis by HPLC, samples were transferred into individual 1.5-mL microcentrifuge tubes and 600 *μ*L high purity Milli-Q water (Milli-Q Integral system, Millipore Corp., Billerica, MA) was added to the first 49 samples and 400 *μ*L Milli-Q water to the remaining 68 samples (a dilution adjustment was made to improve the signal to noise ratio following analysis of the first 49 samples). Within the microcentrifuge tubes, each parasitoid was thoroughly homogenized using a disposable plastic pestle (VWR international Ltd., Lutterworth, UK). The homogenate was then passed through a syringe filter (0.2 *μ*m 17 mm PTFE membrane, Welwyn Garden City, UK) into a 2-mL clear glass wide-necked vial containing a 0.2 mL tapered insert and sealed with a screw cap with an integrated PTFE seal (Chromacol Ltd., Welwyn Garden City, UK). Analyses were conducted as described by Wyckhuys et al. (2008b); 10 *μ*L of each sample was injected into a Dionex ICS 3000 HPLC-system (Dionex Corp., Sunnyvale, CA) equipped with an ICS 3000 dual pump, an Aminotrap Ionpac ATC-3 guard upstream of a 3 × 150 mm CarboPak PA20 analytical column, and an ED 40 electrochemical detector for pulsed amperometric detection (PAD) (Dionex, Leeds, UK). Analytes were separated and eluted at 30°C using a gradient mobile phase comprising 10 and 200 mmol/L NaOH (46–48% HPLC electrochemical grade NaOH solution; Fisher Scientific UK Ltd., Loughborough, UK) at a flow rate of 0.5 mL/min. External calibration standards containing 14 sugars or sugar alcohols (Fructose, Galactose, Glucose, Mannose, Mannitol, Sorbitol, Lactose, Maltose, Sucrose, Trehalose, Erlose, Melezitose, Raffinose, and Stachyose) were prepared in the following concentrations: 2.5, 5.0, 7.5, and 10 ppm. Standards were interspersed between every 10 samples injected to generate calibration curves for quantification and to account for any drift in the performance of the analytical column with repeated sample injection. Peak areas of interest were integrated and quantified with reference to the standards using Chromeleon v6.80 software (Dionex Corp.), and corrections were made for the different sample dilutions.

### Baseline sugar spectra

The nutritional status of field-collected parasitoids were characterized by comparing total sugar levels (i.e., the sum of the concentration of all individual sugars) as well as fructose levels to threshold levels based on a set of laboratory controls. Two sets of laboratory controls were prepared that consisted of newly enclosed (<3 h old) female parasitoids that were transferred into 32-mm-diameter Petri dishes with either *ad libitum* access to water and honeydew (“honeydew-fed”) or water only (“unfed”) for 15 min. Parasitoids were then either immediately frozen at −80°C or kept with water only for another 4 h in a growth chamber (16:8 h light:dark; 60–80% r.h.; 25 ± 5°C) to allow some metabolism of imbibed sugars before also being frozen at −80°C.

Soybean aphid honeydew used for control parasitoids was collected by covering aphid-infested soybean leaves with a piece of Parafilm and storing it in a growth chamber (16:8 h light:dark; 60–80% r.h.; 25 ± 5°C) for 24 h. Each leaf was placed upside down on moist cotton to prevent it from drying out. Honeydew droplets that accumulated on the Parafilm were used immediately to minimize crystallization. This method of honeydew collection was adapted from methods described by Burger et al. (2005) and Wyckhuys et al. (2008b).

In preparation for the HPLC analyses, the ovaries of each control parasitoid were removed and the rest of the body transferred into a microcentrifuge tube filled with 40-*μ*L aqueous Ringer's solution and 100% ethanol (1:1 ratio) and stored at room temperature. Ovaries of control parasitoids were removed to allow a comparison with the equally treated field-collected parasitoids. In addition, 5 *μ*L of pure soybean aphid honeydew was stored in 40 *μ*L Ringer's solution and 100% ethanol (1:1 ratio) and later analyzed using HPLC to obtain the honeydew sugar spectrum.

### Feeding history and nutritional status of field-collected parasitoids

The feeding history of field-collected parasitoids was assessed based on fructose concentrations. Fructose is either not present or present in very low amounts in unfed insects (van Handel 1984; Heimpel et al. 2004; Lee et al. 2004; Steppuhn and Wäckers 2004; Fadamiro and Chen 2005; Hogervorst et al. 2007). Wyckhuys et al. (2008b) showed that fructose levels in sugar-fed female *B. communis* were considerably higher than levels in unfed individuals both immediately after and 4 h after feeding on a sugar source. The nutritional status of field-collected parasitoids was determined using total body sugar levels, which provide a reliable indicator of an insect's feeding status, that is, fed versus unfed (Steppuhn and Wäckers 2004).

Total sugar and fructose levels were log_10_-transformed to meet the assumption of homogeneity and were compared among the three parasitoid groups on the first day after release using ANOVA followed by a Tukey–Kramer HSD test for multiple comparisons of means. The effects of egg load, host density, daily average temperature (°C), hind tibia length, and “morning” versus “afternoon” collection time on total sugar and fructose levels were analyzed using multiple linear regression. Also, a linear regression taking hind tibia length into account was used to assess the effect of fructose and total sugar level on egg load in 2008 (JMP 8.0.1; SAS Institute, Inc., Cary, NC).

## Results

### Host densities, parasitoid recovery rate, and parasitoid fecundity in the field

**Figure 1:**
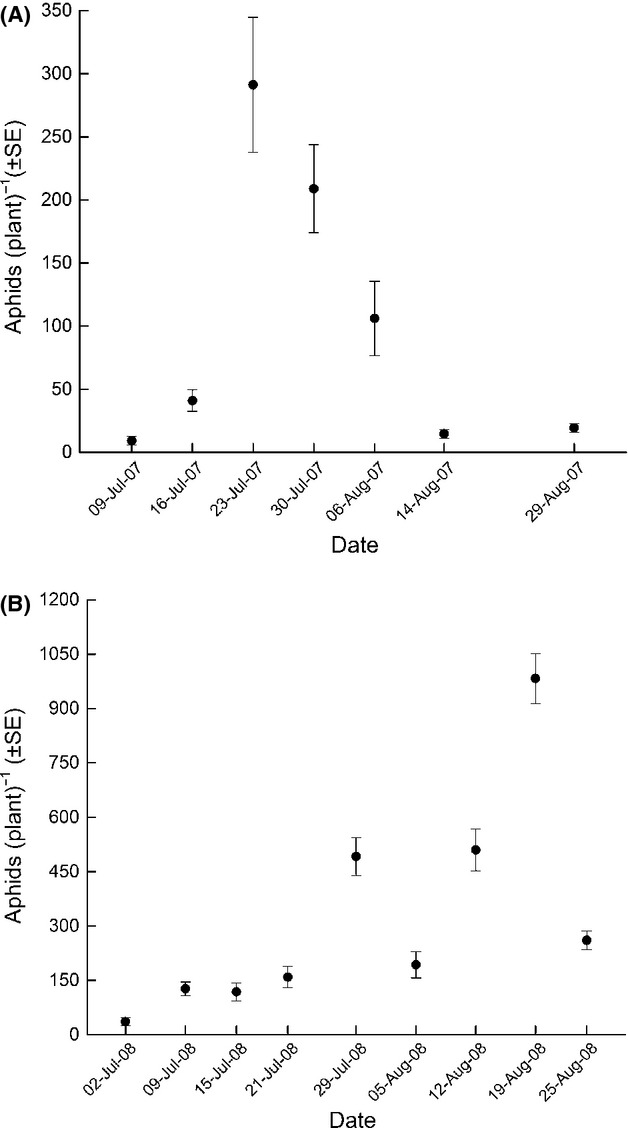
Soybean aphid densities per plant (mean ± SE) by sampling date in (A) 2007 and (B) 2008.

**Figure 2:**
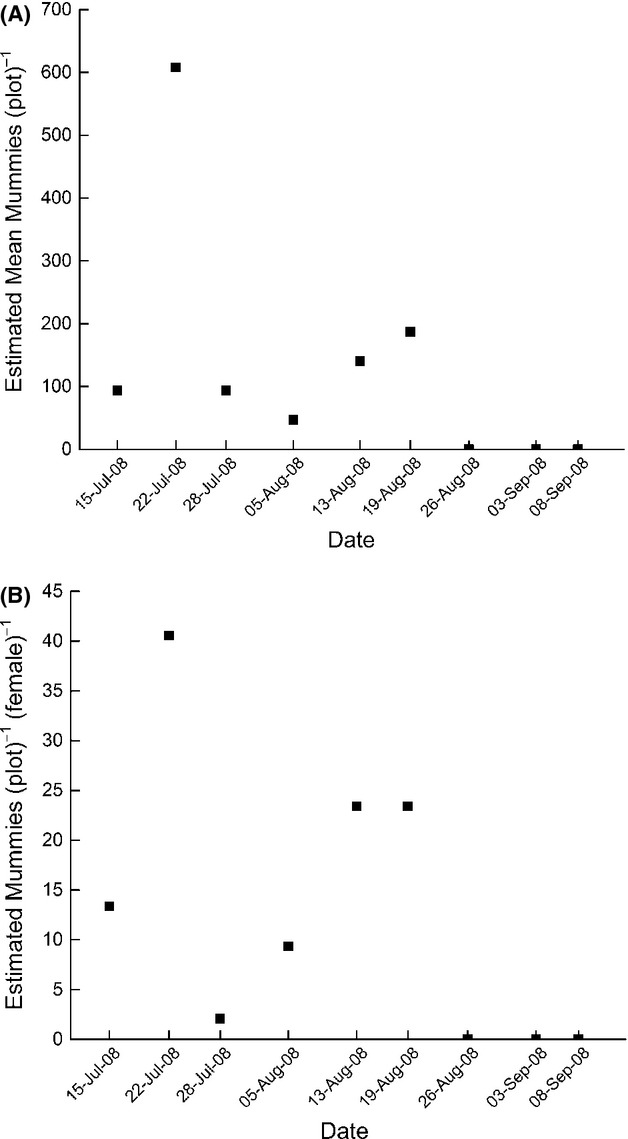
Estimated number of first generation *Binodoxys communis* mummies (A) per plot and (B) per plot and female in 2008.

Host densities ranged on average (±SE) from 9.3 ± 2.4 to 291.2 ± 53.4 soybean aphids per plant in 2007 and 35.9 ± 10.6 to 983.1 ± 68.8 soybean aphids per plant in 2008 (Fig. 1). Per-plant densities were significantly different between sampling dates in 2007 and 2008 (*F*_6,103_ = 39.91, *P* < 0.0001 and *F*_8,81_ = 27.54, *P* < 0.0001, respectively) as well as between the 2 years of this study (*t* = 8.2, df = 198, *P* < 0.0001). In 2008, a total of between 5 and 45 female parasitoids were recovered per plot over the course of the days of postrelease collection. The estimated recapture rate ranged from 0.3 to 6.1% (see also Table S1). The number of first generation *Binodoxys communis* mummies ranged from 0 ± 0 to 1.3 ± 0.4 (±SE) mummies per plant which extrapolates to an estimated 0 to 608 mummies per plot (Fig. 2A). The estimated number of mummies produced per plot per female, that is, fecundity per female, ranged from 0 to 40.6 mummies and was not significantly correlated with sampling date (*F*_1,6_ = 2.87, *P* = 0.1410) (Fig. 2B) or per-plant host density (*F*_1,6_ = 0.11, *P* = 0.7570). There was no significant correlation between the total number of female parasitoids recovered per plot and the estimated number of mummies produced (*F*_1,7_ = 0.37, *P* = 0.5639).

### Egg loads

Egg loads were obtained from 80 field-collected females in 2007 and 117 females in 2008. These egg load data were pooled for the 2 years of collection as there was no significant difference in parasitoid egg load between the 2 years (*F*_1,189_ = 0.38, *P* = 0.5345), or between the 2 years separated by day after release (Year × Day: *F*_1,189_ = 1.87, *P* = 0.1731) or parasitoid group (“morning,” “afternoon” vs “caged”) (Year × Group: *F*_2,189_ = 0.83, *P* = 0.4377).

**Figure 3:**
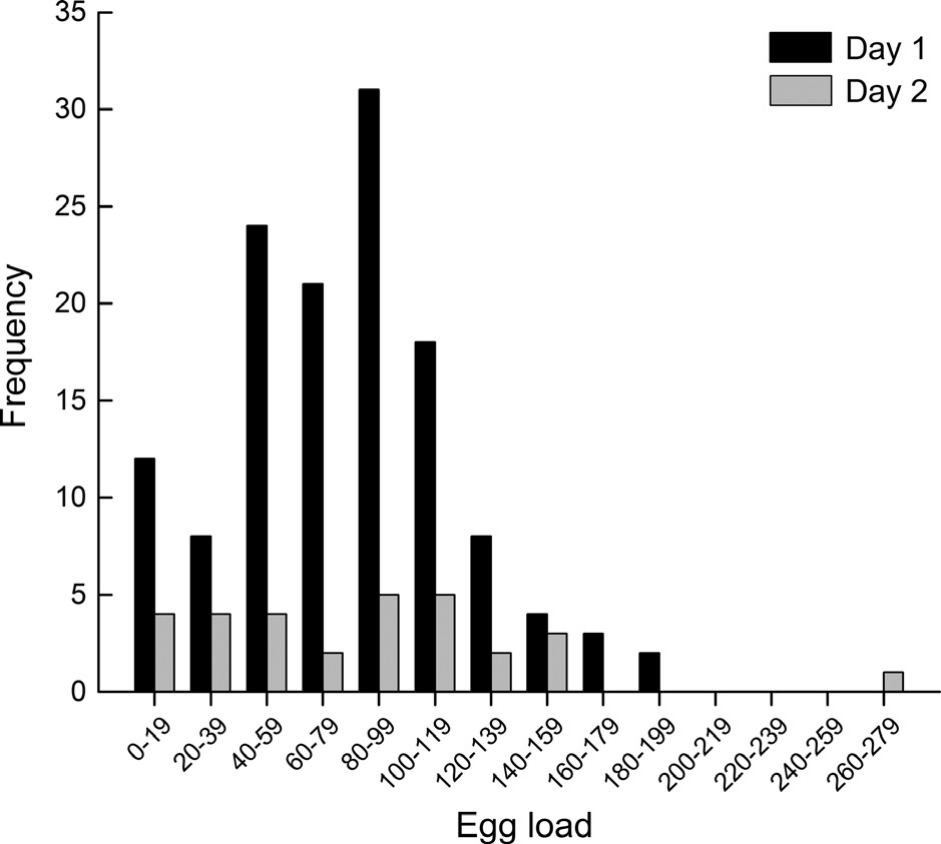
Egg load distribution of 162 “morning” and “afternoon” *Binodoxys communis* females collected in soybean plots in 2007 and 2008 (pooled), separated by day after release.

**Figure 4:**
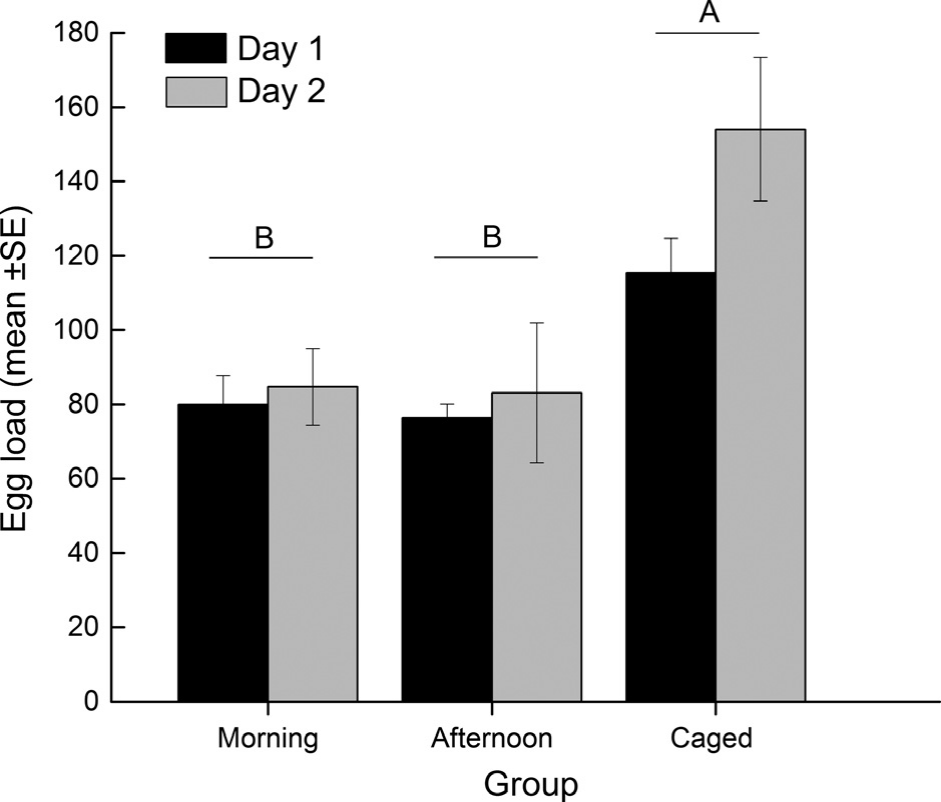
Egg loads (mean ± SE) of “morning,” “‘afternoon,” and “caged” *Binodoxys communis* females, separated by day after release. Bars not connected by the same letter are significantly different (Tukey-Kramer HSD for multiple comparisons, *P* < 0.05).

Egg loads of “morning” and “afternoon” *B. communis* ranged from 1 to 193 eggs on day 1 (*N* = 132) and 6 to 264 eggs on day 2 after release (*N* = 30) (Fig. 3). Average egg loads of those females were 77.77 ± 3.77 eggs (± SE) and 81.70 ± 7.90 eggs (± SE) on days 1 and 2, respectively, and did not differ significantly (*t* = 0.45, df = 160, *P* = 0.6536). Average egg loads of “caged” parasitoids were 115.32 ± 9.34 eggs (± SE; *N* = 28) and 154 ± 19.31 eggs (± SE; *N* = 7) on days 1 and 2, respectively. Egg loads were significantly different among the three parasitoid groups with egg loads of “caged” parasitoids significantly higher than egg loads of “morning” and “afternoon” parasitoids (*F*_2,191_ = 14.15, *P* < 0.0001; Fig. 4).

Calculation of egg maturation rates using egg loads of “morning” and “caged” females collected on days 1 and 2 after release resulted in estimates of 4.36 ± 0.11 eggs per hour matured on day 1 and 8.67 ± 0.67 eggs per hour on day 2. Slopes of egg load over time of day were significantly different from zero on both days (day 1: *F*_1,61_ = 7.4619, *P* = 0.008; day 2: *F*_1,20_ = 12.11, *P* = 0.0024), but a comparison of slopes (Zar 1984) indicated that females did not mature significantly more eggs per hour on day 2 than on day 1 (*t* = 1.29, df = 81, 0.1 < *P* < 0.2). Regression analyses led to estimates that female parasitoids laid on average 3.64 ± 0.85 eggs per hour on day 1 and 7.38 ± 2.15 eggs per hour on day 2. However, these estimated oviposition rates were not significantly different between the 2 days after release (*F*_1,157_ = 0.239, *P* = 0.6256).

**Figure 5:**
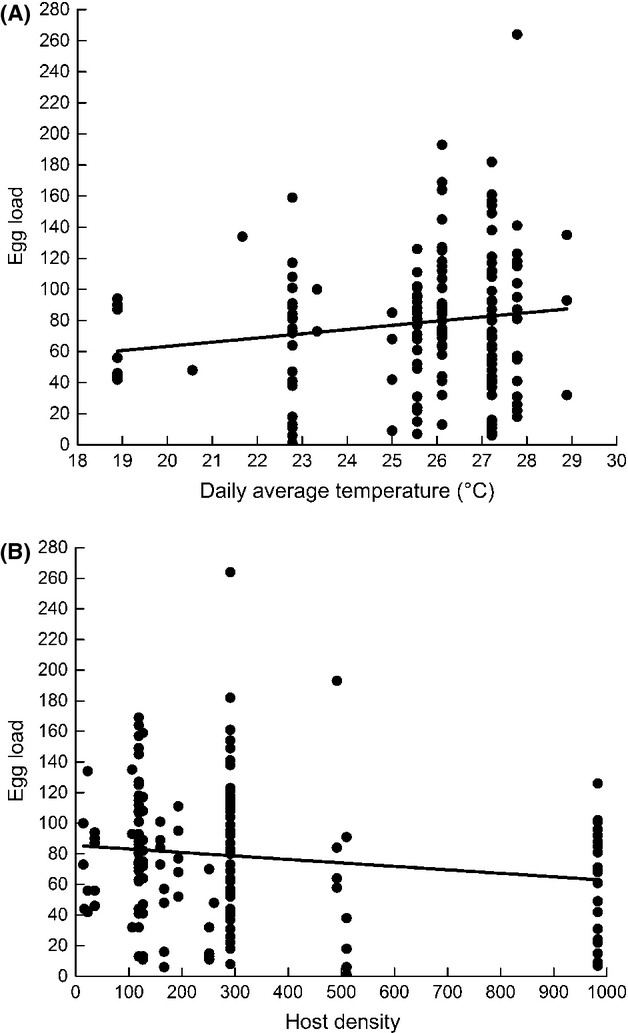
Correlation between egg load and (A) daily average temperature (°C) (linear regression: egg load = 2.708 * average temperature + 9.1769; *r*^2^ = 0.0208) as well as (B) host density; linear regression: egg load = −0.0227 * host density + 85.456; *r*^2^ = 0.0223.

There was a significant positive effect of daily average temperature (*F*_1,156_ = 7.27, *P* = 0.0078; Fig. 5A) and a significant negative effect of host density (*F*_1,156_ = 6.07, *P* = 0.0149; Fig. 5B) on egg load in female *B. communis* collected in the field. However, egg load was not significantly correlated with hind tibia length (*F*_1, 156_ = 0.26, *P* = 0.6084).

### Feeding history and nutritional status in field-collected parasitoids in 2008

Sugar data for 0- and 4-h-old individuals from both the fed and unfed *B. communis* groups in the laboratory were pooled as they were not significantly different from each other. In addition, only sugar levels of *B. communis* collected on the first day post-release were analyzed as very few individuals were collected on the second day. Finally, the sugar spectrum of the pure soybean aphid honeydew sample showed the following sugar composition: Fructose (43.3%), Glucose (19.5%), Erlose (17.4%), Stachyose (14.1%), Sucrose (4.1%), Trehalose (0.9%), and Mannitol (0.8%) (see also Heimpel et al. 2004; Wyckhuys et al. 2008b).

**Figure 6:**
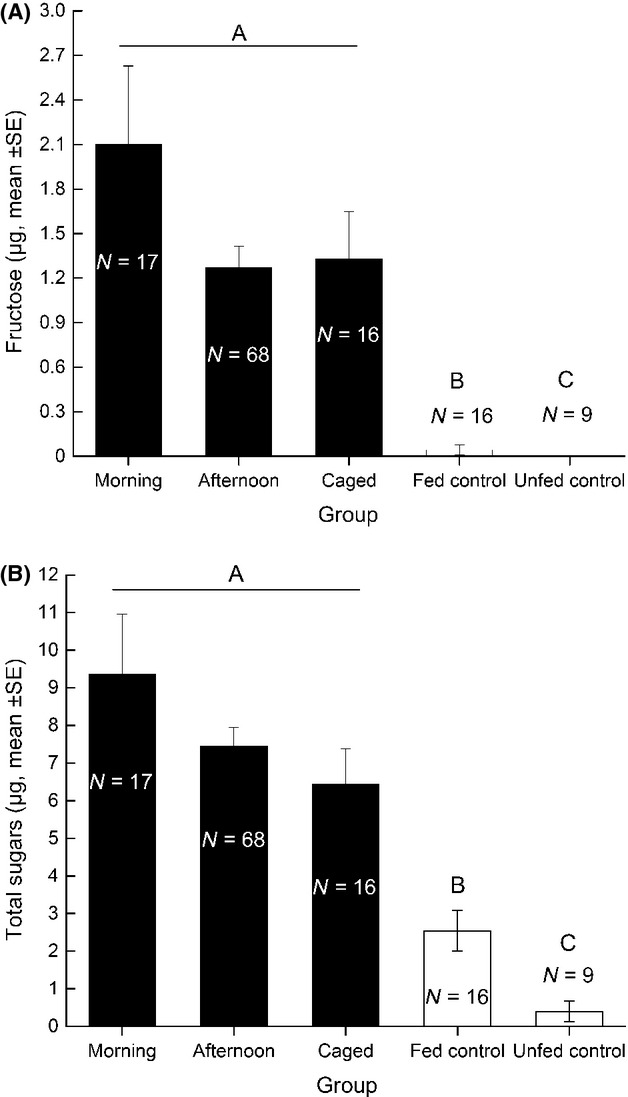
Sugar levels (*μ*g; mean ± SE) of (A) fructose and (B) total sugar in “morning,” “afternoon,” and “caged” *Binodoxys communis* groups on the first day after release (closed bars) as well as the honeydew-fed and starved laboratory controls (open bars). Bars not connected by the same letter are significantly different (Tukey HSD, *P* < 0.05).

No fructose was detected in unfed *B. communis* laboratory controls while 4 of 16 honeydew-fed control *B. communis* did test positive for fructose (0.173 ± 0.12 *μ*g (± SE), *N* = 4). Despite the low fraction of fructose-positive parasitoids in the laboratory controls, the majority of field-collected *B. communis* tested positive for fructose with levels ranging from 0.13 to 9.58 *μ*g. Only a single field-collected individual did not test positive for fructose. Mean fructose levels in the “morning,” “afternoon,” and “caged” groups on the first day after release were all significantly greater than fructose levels of honeydew-fed controls (*F*_3,121_ = 25.42, *P* < 0.0001) (Fig. 6A). Mean total sugar levels of unfed control parasitoids in the laboratory were significantly lower than honeydew-fed ones (0.39 ± 0.28 *μ*g (± SE) versus 2.54 ± 0.54 *μ*g (± SE), *t* = −2.84, df = 23, *P* = 0.0094) (Fig. 6B and the composition of these sugars are reported in Table S2). In all three groups of field-collected parasitoids, mean total sugar levels on the first day after release were significantly higher than total sugar levels of both honeydew-fed and unfed laboratory control parasitoids (*F*_4,121_ = 29.80, *P* < 0.0001), but did not differ significantly from one another (Fig. 6B).

**Figure 7:**
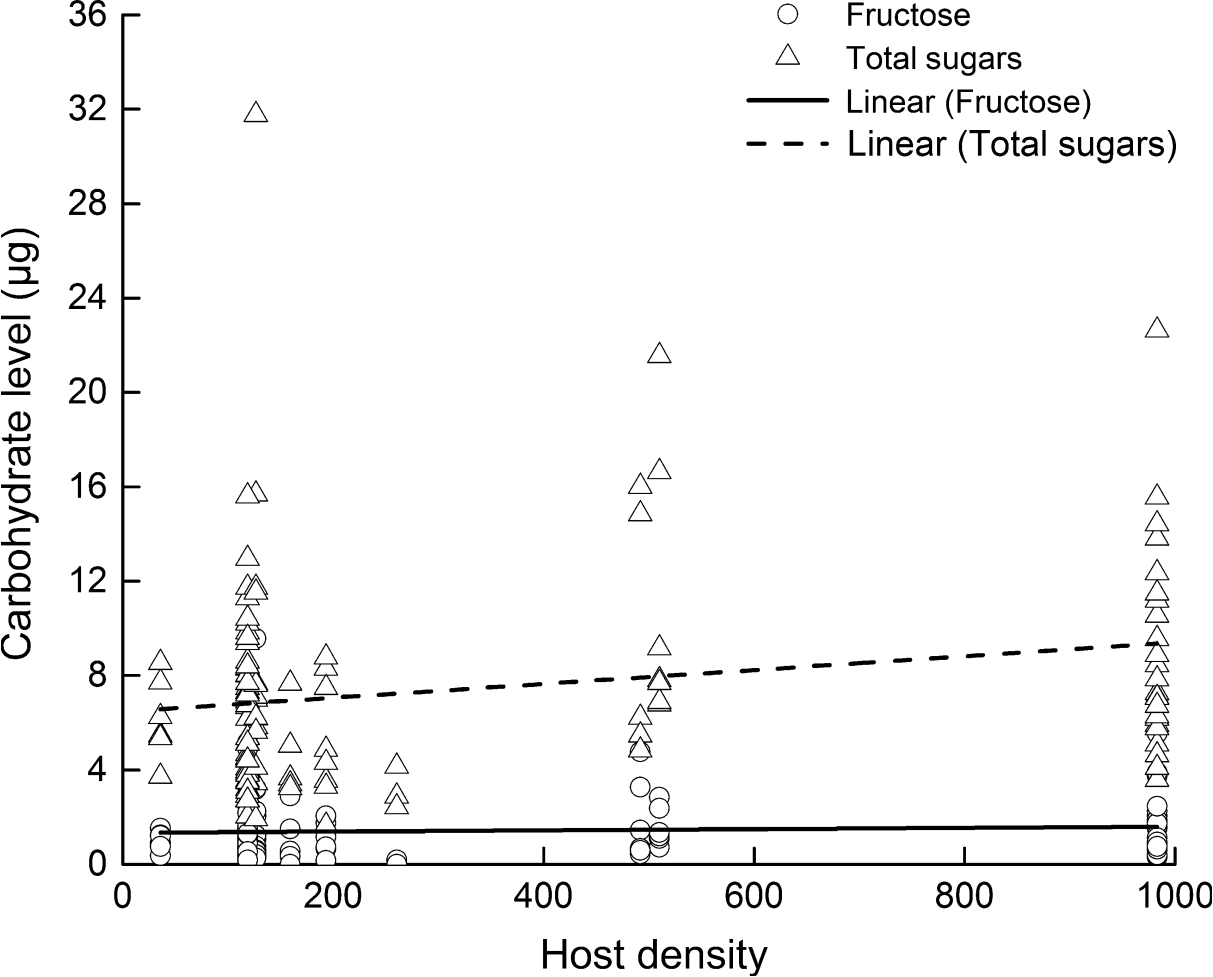
Correlations between host density (log_10_) and levels of total sugars (*μ*g) and fructose (*μ*g) in female *B. communis* collected on the first day after release. Linear regressions: total sugar (*μ*g) = 6.4742*(log10-host density) + 0.0029, *r*^2^ = 0.0511; fructose (*μ*g) = 1.3335*(log10-host density) + 0.0003, *r*^2^ = 0.0041.

In “morning” and “afternoon” *B. communis* collected on the first day after release, both fructose and total sugar levels were significantly correlated with host density (*F*_1,76_ = 4.50, *P* = 0.0372 and *F*_1,89_ = 9.74, *P* = 0.0024, respectively) (Fig. 7). In addition, there was a significant correlation between total sugar levels and hind tibia length (*F*_1,89_ = 11.38, *P* = 0.0011; not shown) but not for fructose levels (*F*_1,76_ = 1.85, *P* = 0.1784). Finally, neither fructose nor total sugar levels were correlated with egg load (*F*_1,76_ = 2.74, *P* = 0.1022 and *F*_1,89_ = 0.18, *P* = 0.6698), daily average temperature (*F*_1,76_ = 0.18, *P* = 0.6749 and *F*_1,89_ = 0.42, *P* = 0.5200), or parasitoid group (*F*_1,76_ = 2.31, *P* = 0.1326 and *F*_1,89_ = 0.19, *P* = 0.6636). The positive effect of host density on both fructose and total sugar levels in *B. communis* suggests that the parasitoids may have fed on hemipteran honeydew in the field prior to their collection.

## Discussion

The majority of female *Binodoxys communis* did not show signs of egg limitation under field conditions. *B. communis* maintained a remarkably constant egg load over time while also maturing an estimated 5.17 ± 1.34 eggs per hour over both days of collections. Furthermore, egg load was inversely correlated with host density suggesting that more eggs were laid in host-rich environments than in host-poor ones. These findings indicate that egg maturation rates offset oviposition with the result that relatively constant egg loads were maintained. Furthermore, there was evidence of sugar-feeding of *B. communis* in the field based on the presence and amount of fructose in almost all field-collected parasitoids. The majority of field-collected parasitoids had high sugar levels indicating that sugar was not a limiting factor in the field. Sugar levels were significantly correlated with host density suggesting that the increased sugar levels were at least partly due to sugar-feeding in the field by *B. communis*.

*Binodoxys communis* maintained a constant egg load of approximately 80 eggs despite significantly positive egg maturation rates estimated during the course of this study. Thus, there is an excess of eggs that are unaccounted for in field-collected parasitoids. Eggs may have been either absorbed (oösorption) or deposited into a nonhost environment (“egg dumping”). In parasitoids, oösorption may function to recycle nutrients (Collier 1995; Rosenheim et al. 2000) or to remove unviable eggs (Rivero-Lynch and Godfray 1997; Asplen and Byrne 2006). In *B. communis,* neither of these functions are likely to have a significant impact on egg load, especially early in its lifetime. First, *B communis*' eggs are rather small and yolk-free (Dieckhoff and Heimpel 2010) and thus are likely a poor source of nutrients (King et al. 1971; Le Ralec 1995). And second, in a previous laboratory study, a decline in egg load was only observed in females older than 48 h (Dieckhoff and Heimpel 2010). In this study, the majority of female *B. communis* was recovered within the first 48 h postemergence. Thus, oösorption might primarily have had an influence on the egg load of the 37 (of 197) parasitoids that were recovered on day 2 after a release as those individuals were around 48–72 h old. Egg dumping can occur when egg maturation is continuous and host densities are too low to provide adequate oviposition opportunities (Quicke 1997; Roberts and Schmidt 2004). It seems highly unlikely, though, that egg dumping would have occurred in freely foraging females that had access to hosts and not in the caged females which were host-deprived and showed a significant increase in egg loads. The possibility that egg maturation rates were increased within the field cages because of an altered microclimate cannot be ruled out but is considered unlikely as well due to abundant ventilation within the cages and the fact that the cages were placed in the shade within the soybean canopy.

The presence of *B. communis* mummies in the field plots suggests that at least some proportion of those eggs was laid in host aphids. Yet based on the estimated fecundity and average egg load per female, there is still an excess of eggs unaccounted for. Egg mortality after oviposition as a result of host defenses and/or susceptibility to parasitism of a host stage can explain the fate of some eggs laid in the field (Wyckhuys et al. 2008a; Desneux et al. 2009a). Also, superparasitism commonly observed in Aphidiines (Chau and Mackauer 1999) or self-superparasitism, which has previously been correlated with egg load and prior oviposition experience (Michaud and Mackauer 1995), may also account for egg mortality within the host. However, *B. communis* uses transient host paralysis as a mechanism to minimize self-superparasitism (Desneux et al. 2009b). Predation may also lead to an underestimation of the number of mummies produced in the field. Insect predators can be abundant in soybean fields (e.g., Costamagna and Landis 2007) and predation on immature stages of *B. communis* has been documented (Chacon and Heimpel 2010; Chacon et al. 2012).

The maintenance of a constant egg load over time suggests that the risk of egg limitation in *B. communis* under field conditions is low, at least within the first 2 days post-eclosion and at the host densities experienced by the parasitoids in the field over the course of this study. We have previously shown that egg maturation in *B. communis* is inversely proportional to current egg load (Dieckhoff and Heimpel 2010). In the laboratory, egg maturation rates increased in a host-rich environment but host contact alone could not explain the observed increase in maturation rates. This suggested that egg maturation rates in *B. communis* respond to the ovarian status of a female by replenishing eggs that have been oviposited (Dieckhoff and Heimpel 2010). Such a response may buffer egg load and therefore lower the risk of becoming egg-limited. This field study supports our laboratory findings that *Binodoxys communis* avoids becoming egg-limited by buffering its egg load.

Despite this buffering, egg load in field-collected *B. communis* declined with increasing host density. To our knowledge, this is the first study showing a negative correlation between egg load and host density. The observed decline in egg load with host density is in accordance with Rosenheim's (1996) prediction that host density should be positively correlated with the proportion of parasitoids that become egg-limited within a population (among populations egg loads may be positively correlated with host density due to selection; Segoli and Rosenheim 2013). One study that characterized egg limitation within a population of field-collected parasitoids found no correlation between egg load and host availability in the armored scale insect parasitoid *Aphytis aonidiae* (Heimpel and Rosenheim 1998). Dynamic host choice behavior in *A. aonidiae* may have contributed to the lack of a relationship between egg load and host availability in that case (Heimpel et al. 1996; Mangel and Heimpel 1998) and in other studies. Conditions of high host density have been directly linked to higher egg maturation rates (Bodin et al. 2009; Casas et al. 2009). A negative effect of host density on egg load implies that parasitoid reproductive success might be constrained by the egg maturation rate under conditions of high host densities (Rosenheim 1996, 1999; Casas et al. 2000; Rosenheim et al. 2000) leading to temporary episodes of egg limitation (Heimpel and Rosenheim 1998; Heimpel et al. 1998; Casas et al. 2000). However, the relationship between host density and egg load in *B. communis*, though significant was relatively weak, with an increase of 100 hosts per plant correlated with a decline in only 2 eggs. The ability of *B. communis* to buffer its egg load clearly contributes to the shallowness of this relationship. Also, the variability in host densities among the 2 years of observation, while being a common occurrence in the Midwestern USA (Ragsdale et al. 2004, 2011), may have played a role in this. By pooling the data for the 2 years of this study, slightly more weight might have been given to egg load under low host density conditions as the first year of the study had a significantly lower host density compared with the second year.

Our data also suggest that *B. communis* are not sugar-limited despite the fact that they were foraging in a homogeneous habitat. The levels of sugar-feeding revealed in this study are higher than most other similar studies of freely foraging parasitoids (Heimpel et al. 2004; Heimpel and Jervis 2005; Hogervorst et al. 2007; Desouhant et al. 2010; Fischbein et al. 2013; Tena et al. 2013), although some other parasitoids have been shown to have similar levels of sugars (Casas et al. 2003; Lavandero et al. 2005; Lee et al. 2006; Rusch et al. 2013). The increased levels of both fructose and total sugars in field-collected *B. communis* strongly suggest that feeding on an exogenous carbohydrate source was a common occurrence in the field. Soybean aphid honeydew was most likely the main source accessible to *B. communis* in the field, and other parasitoid species have been shown to utilize the nectar of this aphid species (Lee et al. 2006). The soybean fields used for our study were highly simplified with a limited diversity of plants that could provide floral or extrafloral nectar. All plots in this study were placed at least 30 m from the field edges and had been treated with Roundup© herbicide, the week prior to the initiation of the study. It is therefore unlikely that females had the opportunity to access a sugar source other than soybean aphid honeydew. Another indication that soybean aphid honeydew was consumed by parasitoids in this study is the positive correlation between host density and sugar levels in field-collected *B. communis*.

Overall concentrations of total body sugars indicate that the majority of female parasitoids had a low risk of starvation in the field, and thus a reduced risk of becoming time-limited due to sugar deprivation. In laboratory studies, female *B. communis* died within 48 h without access to a sugar source but survived up to 3 weeks on a sugar diet (Wyckhuys et al. 2008b; Dieckhoff and Heimpel 2010). Also, Wyckhuys et al. (2008b) showed that female *B. communis* given access to soybean aphid honeydew lived up to three times longer when also given access to water allowing female parasitoids to live up to 9 days on a honeydew+water diet. These authors hypothesized that water increases saliva production in the parasitoid which in turn facilitates the parasitoid's access to crystallizing resources such as honeydew (Stoffolano 1995). Soybean plants in the open field are often covered with dew in the morning and small water pockets can persist in the shaded parts of the canopy for the rest of the day thus forming easily accessible water sources for insects (C. Dieckhoff, pers. obs.). We consider the risk of an individual *B. communis* to become time-limited due to starvation under field conditions to be low. However, other environmental factors such as predation or adverse weather may decrease life expectancy. Insect predators can be abundant in soybean fields (e.g., Costamagna and Landis 2007) and predation on immature stages of *B. communis* has been documented (Chacon and Heimpel 2010; Chacon et al. 2012). The risk of predation on adult *B. communis* in the field has not been addressed, but other studies have indicated high levels of predation on adult parasitoids in the field (Heimpel et al. 1997). Finally, rainfall has been shown to induce substantial mortality in insects (Moran and Hoffmann 1987; Roitberg et al. 1992; Weisser et al. 1997). Thus, both predation and weather conditions have the potential to increase the risk of time limitation in *B. communis* under field conditions.

In conclusion, the majority of field-collected female *B. communis* were not egg-limited under field conditions. Female parasitoids maintained a constant egg load over time, and we hypothesize that this process might provide a buffer to egg load, and thus reduce the risk of becoming egg-limited. Such a buffering mechanism may be adaptive in environments with variable host densities (Sevenster et al. 1998) and this as well as a low risk of starvation may result in greater host suppression (Getz and Mills 1996; Schreiber 2007).

## Supporting Information

**Table S1.** Sex ratios, numbers of adult *Binodoxys communis*, and estimated numbers of female *B. communis* released per container and plot as well as total number of recaptured female *B. communis* and estimated recapture rate. Data for 2008 only.

**Table S2.** Micrograms (± SEM) of 12 sugars and 2 sugar alcohols from HPLC analyses of 117 *B. communis* individuals field collected in 2008.
