# Supplementary material for: Egg load dynamics and the risk of egg and time limitation experienced by an aphid parasitoid in the field
Source: Ecol Evol. 2014 Apr 11;4(10):1739–50. doi: 10.1002/ece3.1023 (PMC4063472; doi:10.1002/ece3.1023)
Supplement: Supplementary file 1 [file ece30004-1739-SD1.doc]

**Supplementary Table 1**. Sex ratios, numbers of adult *Binodoxys communis* and estimated numbers of female *B. communis* released per container and plot as well as total number of recaptured female *B. communis* and estimated recapture rate. Data for 2008 only.

|  | Container 1 | | |  | Container 2 | | |  | Container 3 | | |  | Plot | | |
| --- | --- | --- | --- | --- | --- | --- | --- | --- | --- | --- | --- | --- | --- | --- | --- |
| Release Date | Sex Ratio | No. adult parasitoids Released | Estimated No. female parasitoids Released |  | Sex Ratio | No. adult parasitoids Released | Estimated No. female parasitoids Released |  | Sex Ratio | No. adult parasitoids Released | Estimated No. female parasitoids Released |  | Total No. female parasitoids Released | Total No. female parasitoids Recaptured | Recapture Rate |
| 2-Jul | 0.60 | 88 | 35.55 |  |  |  |  |  | 0.50 | 160 | 79.86 |  | 115.41 | 7 | 0.061 |
| 9-Jul | 0.46 | 341 | 183.43 |  | 0.35 | 235 | 151.67 |  | 0.22 | 410 | 321.17 |  | 656.27 | 15 | 0.023 |
| 15-Jul | 0.52 | 952 | 458.57 |  | 0.59 | 719 | 293.74 |  | 0.46 | 795 | 432.89 |  | 1185.20 | 45 | 0.038 |
| 21-Jul | 0.53 | 135 | 63.32 |  | 0.57 | 381 | 165.35 |  | 0.47 | 104 | 54.91 |  | 283.58 | 5 | 0.018 |
| 29-Jul | 0.20 | 166 | 133.46 |  | 0.45 | 153 | 83.84 |  | 0.43 | 311 | 177.89 |  | 395.20 | 6 | 0.015 |
| 5-Aug | 0.58 | 621 | 260.20 |  | 0.58 | 865 | 362.44 |  | 0.57 | 921 | 392.35 |  | 1014.98 | 8 | 0.008 |
| 12-Aug | 0.56 | 274 | 120.83 |  | 0.47 | 330 | 174.57 |  | 0.36 | 336 | 216.38 |  | 511.79 | 7 | 0.014 |
| 19-Aug | 0.40 | 434 | 259.53 |  | 0.55 | 767 | 342.85 |  | 0.57 | 655 | 279.69 |  | 882.07 | 26 | 0.029 |
| 25-Aug | 0.59 | 1300 | 533.00 |  | 0.47 | 1205 | 641.06 |  | 0.47 | 1440 | 757.44 |  | 1931.50 | 5 | 0.003 |
