# Supplementary material for: Egg load dynamics and the risk of egg and time limitation experienced by an aphid parasitoid in the field
Source: Ecol Evol. 2014 Apr 11;4(10):1739–50. doi: 10.1002/ece3.1023 (PMC4063472; doi:10.1002/ece3.1023)
Supplement: Supplementary file 2 [file ece30004-1739-SD2.doc]

**Supplementary Table 2.** Micrograms (± SEM) of 12 sugars and 2 sugar alcohols from HPLC analyses of 117 *B. communis* individuals field collected in 2008.

| Fructose | Galactose | Glucose | Mannose | Mannitol | Sorbitol | Lactose | Maltose | Sucrose | Trehalose | Erlose | Melezitose | Raffinose | Stachyose |
| --- | --- | --- | --- | --- | --- | --- | --- | --- | --- | --- | --- | --- | --- |
| 1.423  (0.129) | 0.023  (0.012) | 2.956  (0.211) | 0.002  (0.001) | 0.035  (0.005) | 0.027  (0.009) | 0.000  (0.000) | 0.049  (0.027) | 2.414  (0.249) | 0.275  (0.027) | 0.252  (0.073) | 0.004  (0.003) | 0.014  (0.014) | 0.015  (0.012) |
